# Supplementary material for: uRP: An integrated research platform for one-stop analysis of medical images
Source: Front Radiol. 2023 Apr 18;3:1153784. doi: 10.3389/fradi.2023.1153784 (PMC10365282; doi:10.3389/fradi.2023.1153784)
Supplement: Supplementary file 1 [file Table1.docx]

Supplementary Material

**Supplementary Table 1.** **A total of 2,264 radiomics features are generated from one specific image.** The original image is transformed with 24 image filters to generate additional 24 images, in which Wavelet filter owns 8 cases (i.e., HHH, HHL, HLH, LHH, HLL, LHL, LLH, LLL) and Laplacian of Gaussian filter owns 4 cases (i.e., sigma 0.5 mm, 1.0 mm, 1.5 mm, 2.0 mm). For each filter, features are computed for seven categories, including histogram, GLCM, GLRLM, GLSZM, NGTDM, GLDM, and shape.

| Filter  Category | Histogram | GLCM | GLRLM | GLSZM | NGTDM | GLDM | Shape |
| --- | --- | --- | --- | --- | --- | --- | --- |
| Original image | 18 | 21 | 16 | 16 | 5 | 14 | 14 |
| Shot noise | 18 | 21 | 16 | 16 | 5 | 14 | - |
| Speckle noise | 18 | 21 | 16 | 16 | 5 | 14 | - |
| Additive Gaussian noise | 18 | 21 | 16 | 16 | 5 | 14 | - |
| Binomial blur image | 18 | 21 | 16 | 16 | 5 | 14 | - |
| Mean filter | 18 | 21 | 16 | 16 | 5 | 14 | - |
| Normalize filter | 18 | 21 | 16 | 16 | 5 | 14 | - |
| Box mean filter | 18 | 21 | 16 | 16 | 5 | 14 | - |
| Box signal image | 18 | 21 | 16 | 16 | 5 | 14 | - |
| Recursive Gaussian | 18 | 21 | 16 | 16 | 5 | 14 | - |
| Discrete Gaussian | 18 | 21 | 16 | 16 | 5 | 14 | - |
| Wavelet (8) | 144 | 168 | 128 | 128 | 40 | 112 | - |
| Laplacian of Gaussian (4) | 72 | 84 | 64 | 64 | 20 | 56 | - |
| Laplacian sharpening | 18 | 21 | 16 | 16 | 5 | 14 | - |
| Curvature flow | 18 | 21 | 16 | 16 | 5 | 14 | - |
